# Underwater peroral endoscopic myotomy improves visualization and reduces adverse events in a high-risk patient with type III achalasia

**DOI:** 10.1055/a-2721-8976

**Published:** 2025-10-29

**Authors:** Paolo Cecinato, Angelo Bruni, Liboria Laterza, Daniele Mandolesi, Michele Dota, Stefania Cappetta, Giovanni Barbara

**Affiliations:** 1 18508 Gastroenterology Unit, IRCCS Azienda Ospedaliero-Universitaria di Bologna, Bologna, Italy; 2 198207 University of Bologna, Department of Medical and Surgical Sciences Bologna, Bologna, Italy; 3 19001 Department of Internal Medicine and Medical Therapy Pavia, University of Pavia, Pavia, Italy

Underwater peroral endoscopic myotomy (uPOEM), a variation of peroral endoscopic myotomy (POEM), eliminates gas insufflation, reducing complications such as pneumomediastinum and capnothorax, particularly in high-risk patients with comorbidities like chronic obstructive pulmonary disease (COPD 1 2 ). The underwater approach improves visualization of tissue planes, enhances the dissection process, and facilitates vessel cauterization, minimizing adverse effects 3 4 .

An 84-year-old man with type III achalasia (Eckardt score: 9) and severe COPD and other comorbidities presented with severe dysphagia with regurgitation and malnutrition was referred for POEM. Given his high risk for insufflation-related complications, uPOEM was performed under general anesthesia with the patient in the supine position. Using a single-channel Olympus gastroscope equipped with a distal cap and a 4 mm T-type gold-knife (Micro-Tech, Nanjing, China), a mucosal incision (endocut-I) was made on the posterior esophageal wall at 23 cm. Submucosal tunneling (endocut-Q or precise SECT) was then carried out under continuous saline immersion, extending to 43 cm, thus entering the gastric side ( Fig. 1 ). Subsequently, a selective myotomy (endocut-Q) of the circular muscle fibers was performed from 24 to 42 cm, also using the underwater technique. The saline immersion significantly enhanced visualization due to water-induced magnification, allowing for clearer differentiation of submucosal and muscular layers and improving vessel identification and cauterization ( Fig. 2 ; Video 1 ). However, a mild reduction in cutting efficiency, attributed to the saline environment, necessitated adjustments to electrosurgical settings. No adverse events occurred during or after the procedure. Barium swallow performed 14 days after the procedure confirmed the complete passage of barium.

**Fig. 1:**
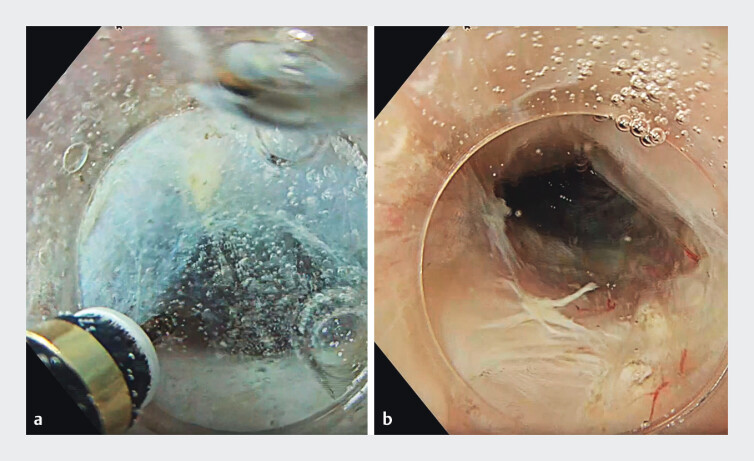
Underwater submucosal tunneling. **a** Mucosal incision on the posterior esophageal wall under continuous saline immersion. **b** Submucosal tunnel extended to the gastric side.

**Fig. 2:**
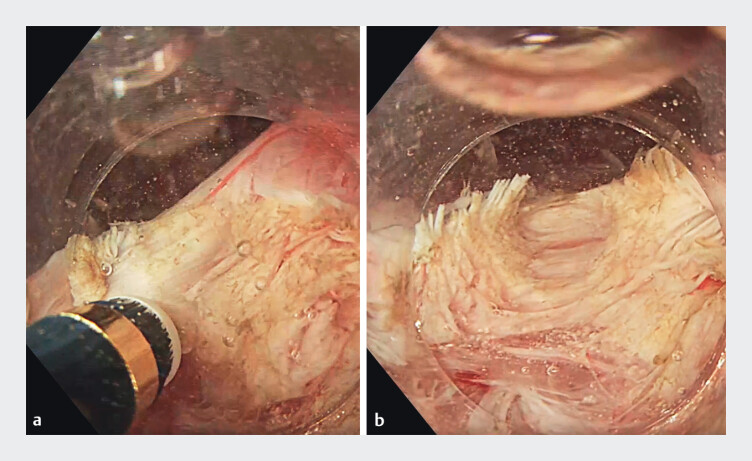
Underwater selective myotomy. **a** Initial dissection of the circular muscle fibers from 24 cm to 42 cm using the underwater technique. **b** Clear delineation of the submucosal and muscular layers facilitated by the saline environment.

Underwater peroral endoscopic myotomy (uPOEM) in a high-risk patient with type III achalasia and severe chronic obstructive pulmonary disease (COPD).Video 1

Studies have demonstrated the feasibility and safety of uPOEM, with high clinical success rates and minimal complications 3 . This approach is particularly beneficial to patients with insufflation-related risk factors, as it reduces adverse events and improves procedural outcomes 2 4 . Standardized protocols further optimize its efficacy, highlighting its role as a valuable alternative to conventional POEM in selected cases 1 5 .

uPOEM effectively combines safety and procedural precision, making it an ideal technique for managing achalasia in high-risk patients while minimizing complications and enhancing surgical outcomes.

Endoscopy_UCTN_Code_TTT_1AO_2AP

## Notes

Endoscopy E-Videos https://eref.thieme.de/e-videos
*E-Videos* is an open access online section of the journal *Endoscopy* , reporting on interesting cases and new techniques in gastroenterological endoscopy. All papers include a high-quality video and are published with a Creative Commons CC-BY license. Endoscopy E-Videos qualify for HINARI discounts and waivers and eligibility is automatically checked during the submission process. We grant 100% waivers to articles whose corresponding authors are based in Group A countries and 50% waivers to those who are based in Group B countries as classified by Research4Life (see: https://www.research4life.org/access/eligibility/ ). This section has its own submission website at https://mc.manuscriptcentral.com/e-videos .
